# Carrying-Over Effects of GVBD Blocking on Post-Blocking Meiotic Progression of Oocytes: Species Difference and the Signaling Pathway Leading to MPF Activation

**DOI:** 10.1371/journal.pone.0103838

**Published:** 2014-07-31

**Authors:** Guang-Zhong Jiao, Hua-Yu Lian, Yan Gao, Ming-Ju Sun, Shuai Gong, Liang-Liang Zheng, Chuan-Xin Zhang, Jing-He Tan

**Affiliations:** College of Animal Science and Veterinary Medicine, Shandong Agricultural University, Tai-an City, P. R. China; Institute of Zoology, Chinese Academy of Sciences, China

## Abstract

Efforts to improve the quality of in vitro matured oocytes by blocking germinal vesicle breakdown (GVBD) and allowing more time for ooplasmic maturation have achieved little due to a lack of knowledge on the molecular events during GVBD blocking. Such knowledge is also important for studies aimed at regulating gene expression in maturing oocytes prior to GVBD. We studied species difference and signaling pathways leading to the carrying-over effect of GVBD blocking on post-blocking meiotic progression (PBMP). Overall, GVBD-blocking with roscovitine decelerated PBMP of mouse oocytes but accelerated that of pig oocytes. During blocking culture, whereas cyclin B of pig oocytes increased continuously, that of mouse oocytes declined first and then increased slowly. In both species, (a) whereas active CDC2A showed a dynamics similar to cyclin B, inactive CDC2A decreased continuously; (b) when oocytes were blocked in blocking medium containing cycloheximide, PBMP was decelerated significantly while cyclin B and active CDC2A decreasing to the lowest level; (c) whereas sodium vanadate in blocking medium reduced PBMP, epidermal growth factor (EGF) in blocking medium accelerated PBMP significantly with no effect on cyclin B levels. In conclusion, the EGF signaling cascade accelerated PBMP by promoting the pre-MPF (M-phase-promoting factor) to MPF conversion during GVBD blocking with roscovitine. The significant difference in PBMP observed between mouse and pig oocytes was caused by species difference in cyclin B dynamics during blocking culture as no species difference was observed in either pre-MPF to MPF conversion or the EGF signaling activity.

## Introduction

The developmental capacity of in vitro matured oocytes is markedly lower than that of their in vivo matured counterparts [1], [2]. Increasing evidence demonstrates that oocyte quality depends on the events before germinal vesicle (GV) breakdown (GVBD), suggesting that the oocyte must accumulate the appropriate information for meiotic resumption, fertilization and embryo development before chromosome condensation [3]. Thus, in vivo, oocytes acquire cytoplasmic maturity after a long series of preparatory processes involving transcription and translation of transcripts during the meiotic prophase [4], [5], whereas in vitro, a premature GVBD without adequate cytoplasmic maturation is induced by transfer of oocytes from follicles into culture medium. To improve the quality of in vitro matured oocytes, attempts have been made to increase the ooplasmic maturation by temporary inhibition of meiotic resumption [6]–[8]. However, little has been achieved in this area; the best inhibition protocols developed so far could only avoid compromising, not to mention improvement on oocyte developmental potential [9], [10]. Therefore, meticulous studies are needed to explore the molecular events during GVBD inhibition to establish an efficient inhibition system for the improvement of ooplasmic maturation. Furthermore, many procedures aimed at regulating gene expression of maturing GV oocytes such as gene knockdown or over expression and RNA interference often involve oocyte culture with meiosis arrested, and such procedures will definitely benefit from knowledge on the molecular events during GVBD blocking.

A carrying-over effect on meiotic progression has been observed following drug inhibition of oocyte GVBD in different species. For example, porcine oocytes treated with roscovitine underwent an accelerated meiotic progression after removal of this meiotic inhibitor; the oocytes reached the MII stage faster than untreated control oocytes [10]. The GVBD of bovine oocytes were also accelerated after treatment with butyrolactone I or roscovitine for GVBD blocking [9], [11]. However, our previous study [12] and unpublished dada have indicated that the meiotic progression of mouse oocytes is decelerated after GVBD blocking with either cycloheximide or roscovitine. The mechanisms for this species-related carrying-over effect of GVBD inhibition are unknown, but may suggest different pathways of metabolism and/or signaling during inhibition. Thus, work on this issue will definitely contribute to our understanding of the molecular events during GVBD inhibition.

A large body of evidence has demonstrated that the M-phase promoting factor (MPF) plays a key role in the resumption of meiosis. The MPF is a complex consisting of a catalytic subunit CDC2A and a regulatory subunit cyclin B [13]. The activation of CDC2A involves two levels. The first level is its association with cyclin B, whose synthesis and degradation oscillates during the cell cycle [14]. The second level entails the phosphorylation and de-phosphorylation of different sites on CDC2A itself. The phosphorylation of CDC2A on T14 and Y15 by the Myt1 and Wee1 kinases produces an inactive form of MPF called pre-MPF [15]. For full activation, CDC2A needs to be phosphorylated on T161 by CDC-activating kinase (CAK) and dephosphorylated on T14 and Y15 by cdc25 phosphatase [16]. However, it is not known whether the drugs used for blocking GVBD of oocytes produce their carrying-over effects of accelerating or decelerating the post-blocking meiotic progression (PBMP) by affecting cyclin B synthesis/degradation, pre-MPF formation or conversion of pre-MPF to MPF.

Epidermal growth factor (EGF) receptor is a tyrosine kinase [17], [18] and when activated, it induces the phosphorylation of serine/threonine residues on a number of proteins, suggesting that multiple serine/threonine kinases may be activated as part of the EGF signaling cascade [19]. In addition, the activation of EGF receptor has been found leading to a rapid resumption of meiosis [20], [21]. However, although this suggests that an EGF-like stimulation is involved in the activation of CDC2A, direct evidences are limited on the role of the EGF signaling cascade in activating CDC2A of the oocyte. Furthermore, we do not know whether the EGF receptor is activated during GVBD inhibition and whether its activation would lead to CDC2A activation and thence the carrying-over effect after GVBD inhibition.

In summary, although efforts have been made to improve the quality of in vitro matured oocytes by blocking GVBD and allowing more time for ooplasmic maturation, little has been achieved in this area due to a lack of full understanding of the molecular events during GVBD blocking. Procedures aimed at regulating gene expression of GV oocytes will also benefit from knowledge on the molecular events during GVBD blocking, because such procedures often involve oocyte culture with meiosis arrested. Furthermore, although a carrying-over effect on meiotic progression has been observed following GVBD blocking, the underlying mechanisms are unknown. The objective of this study was to investigate the species differences and the signaling events leading to the carrying-over effect. Emphasis was placed on the cyclin B dynamics, the pre-MPF to MPF conversion and the EGF signaling activity during the blocking culture.

## Results

### Blocking GVBD with Roscovitine decelerated PBMP of mouse oocytes but accelerated that of pig oocytes

**Figure 1:**
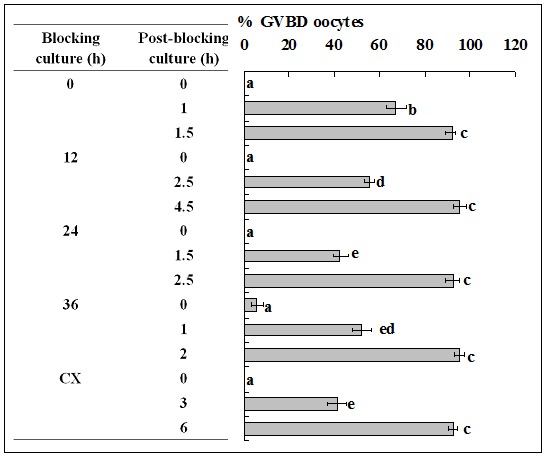
Nuclear progression of mouse oocytes following GVBD blocking for different times with roscovitine. Each treatment was repeated 3 times and each replicate contained some 20 oocytes. CX: Oocytes that had been blocked with roscovitine for 12 h were further treated for 12 h with roscovitine plus CHX to inhibit protein synthesis. a–e: Values without a common letter in their bars differ (P<0.05).

**Figure 2:**
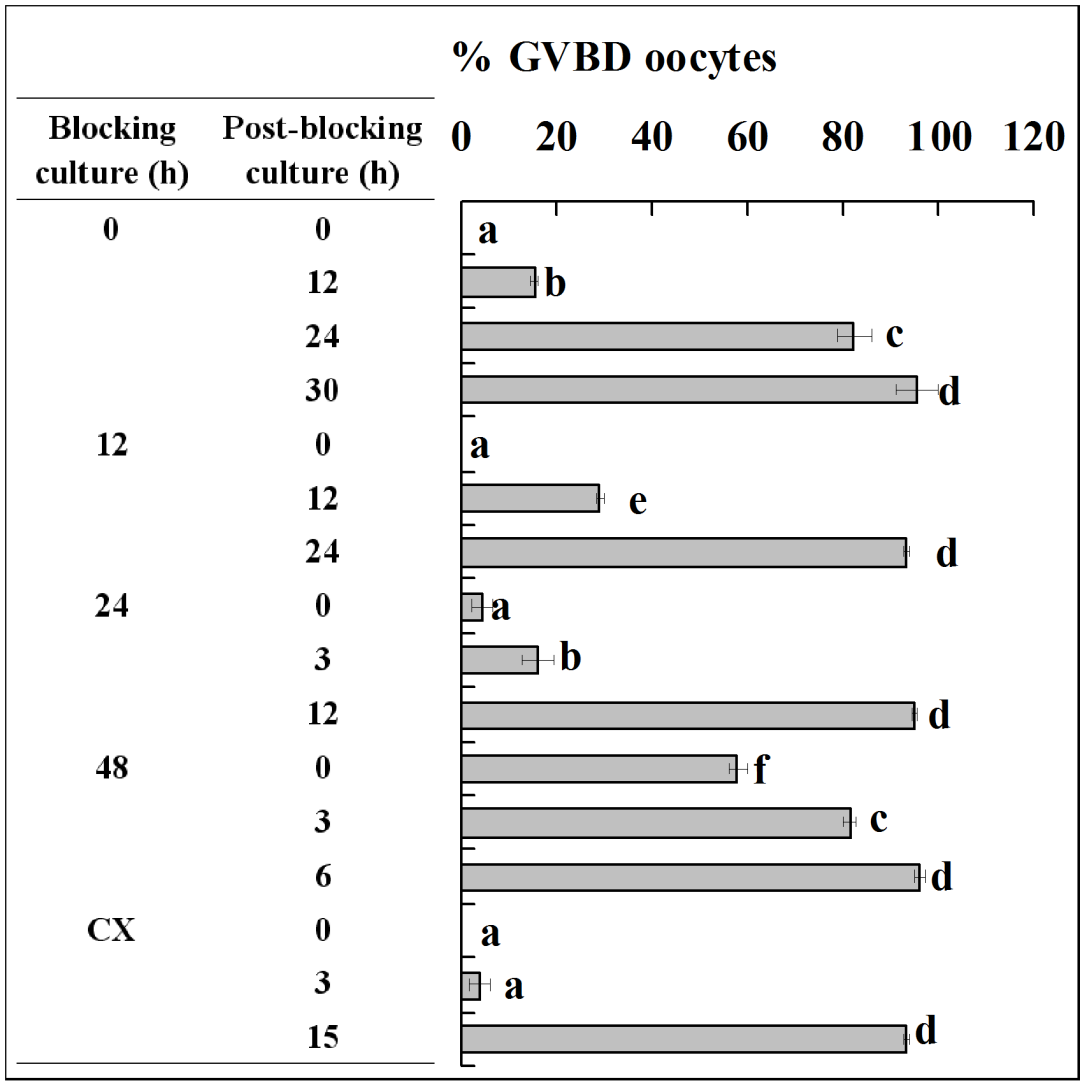
Nuclear progression of pig oocytes following GVBD blocking for different times with roscovitine. Each treatment was repeated 4–5 times and each replicate contained 15–20 oocytes. CX: Oocytes were blocked for 24 h with roscovitine plus CHX to inhibit protein synthesis. a–f: Values without a common letter in their bars differ (P<0.05).

Mouse and pig oocytes were blocked in blocking medium before post-blocking culture with basic culture medium. Oocytes were examined for GVBD at different times of the post-blocking culture. Whereas the non-blocked mouse oocytes completed GVBD within 1.5 h, mouse oocytes blocked for 12, 24 and 36 h did not complete GVBD until 4.5, 2.5 and 2 h of post-blocking culture, respectively (Fig. 1). Whereas the non-blocked pig oocytes completed GVBD at 30 h, pig oocytes blocked for 12, 24 and 48 h completed GVBD at 24, 12 and 6 h of post-blocking culture, respectively (Fig. 2).

### Western blot analysis of cyclin B, total CDC2A, inactive and active P-CDC2A following blocking culture of mouse and pig oocytes

**Figure 3:**
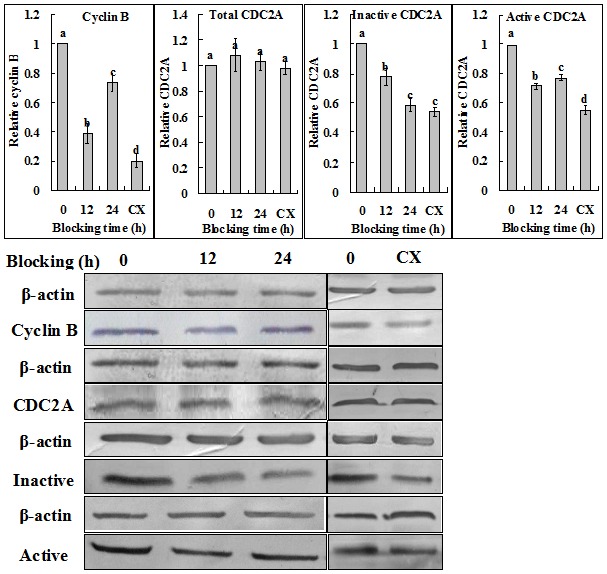
Western blot analysis for levels of Cyclin B, total CDC2A, inactive and active p-CDC2A in mouse oocytes following GVBD blocking for different times with roscovitine. CX: Oocytes that had been blocked with roscovitine for 12 h were further treated for 12 h with roscovitine plus CHX to inhibit protein synthesis. a–d: Values without a common letter in their bars differ (P<0.05).

**Figure 4:**
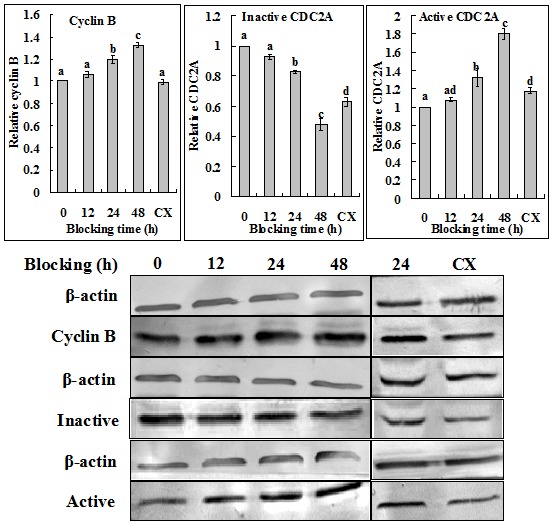
Western blot analysis for levels of Cyclin B, inactive and active p-CDC2A in pig oocytes following GVBD blocking for different times with roscovitine. CX: Oocytes were blocked for 24 h with roscovitine plus CHX to inhibit protein synthesis. a–d: Values without a common letter in their bars differ (P <0.05).

Mouse and pig oocytes were blocked in respective blocking medium, and at different times of the blocking culture, Western analysis was conducted for cyclin B, total CDC2A, inactive and active p-CDC2A. During blocking culture of mouse oocytes, whereas both cyclin B and active p-CDC2A first decreased to the lowest level at 12 h, and then increased to some extent, the level of inactive p-CDC2A underwent a continuous decrease (Fig. 3). This suggested that cyclin B degraded with no or little synthesis up to 12 h of blocking culture, but after that, its synthesis began or surpassed its degradation in mouse oocytes. During blocking culture of pig oocytes, whereas both cyclin B and active p-CDC2A increased continuously, the level of inactive p-CDC2A underwent a continuous decrease (Fig. 4). This suggested that in pig oocytes, cyclin B was synthesized continuously with no or little degradation during blocking culture. The level of total CDC2A did not change during the whole period of blocking culture of either mouse or pig oocytes.

### Inhibiting protein synthesis during blocking culture decelerated PBMP by down regulating cyclin B expression

When mouse oocytes that had been blocked with roscovitine for 12 h were further treated for 12 h with roscovitine plus CHX to inhibit protein synthesis, time for GVBD completion was dramatically postponed to 6 h of post blocking culture (Fig. 1), and levels of cyclin B, inactive and active p-CDC2A all decreased to the lowest level (Fig. 3). When pig oocytes were blocked for 24 h with roscovitine plus CHX, time for GVBD completion was dramatically postponed to 15 h of post blocking culture (Fig. 2), and levels of cyclin B, inactive and active p-CDC2A all decreased significantly, compared to those in oocytes blocked for 24 h without CHX (Fig. 4). CHX in blocking medium showed no effect on the level of total CDC2A in either mouse (Fig. 3) or pig oocytes (data not shown).

### Analysis on the dynamics of the MPF components during blocking culture and its correlation with PBMP

Taken together, the above results suggested the following. Firstly, blocking GVBD with roscovitine decelerated or accelerated PBMP by down or up regulating the level of cyclin B, respectively. Thus, GVBD completion was most postponed after mouse oocytes were blocked for 12 h in roscovitine (Fig. 1) when cyclin B decreased to the lowest level (Fig. 3); GVBD blocking with roscovitine accelerated PBMP of pig oocytes (Fig. 2) with increasing levels of cyclin B (Fig. 4); and treatment with CHX inhibited cyclin B synthesis while postponing PBMP dramatically in both species. Secondly, the negative correlation between inactive and active CDC2A suggested an active conversion of pre-MPF to MPF during the blocking culture. Thus, whereas inactive CDC2A decreased, the active CDC2A increased continuously up to 48 h of blocking culture of pig oocytes (Fig. 4). A similar negative correlation was also observed in mouse oocytes from 12 h to 24 h of blocking culture (Fig. 3). Furthermore, the dramatic decrease in inactive CDC2A along with the same extent of increase in active CDC2A at 48 h of blocking culture suggested a burst of pre-MPF to MPF conversion when oocytes began GVBD, because some 60% of the pig oocytes underwent GVBD by this time (Fig. 2).

### Treatment with Sodium Vanadate decelerated PBMP of Both Mouse and pig oocytes

**Figure 5:**
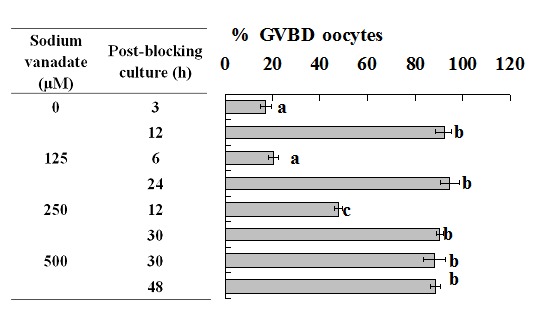
Nuclear progression of pig oocytes after GVBD blocking for 24 h with roscovitine in the presence of sodium vanadate at different concentrations. Each treatment was repeated 4–5 times and each replicate contained 15–20 oocytes. a–c: Values without a common letter in their bars differ (P<0.05).

**Figure 6:**
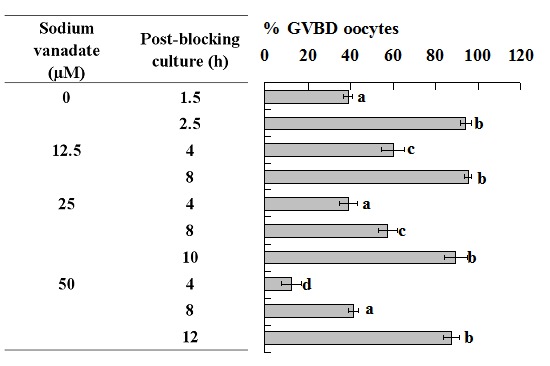
Nuclear progression of mouse oocytes after GVBD blocking for 24 h with roscovitine in the presence of sodium vanadate at different concentrations. Each treatment was repeated 3 times and each replicate contained some 20 oocytes. a–d: Values without a common letter in their bars differ (P<0.05).

To confirm the pre-MPF to MPF conversion during blocking culture, pig and mouse oocytes were blocked for 24 h with roscovitine and different concentrations of sodium vanadate to inhibit the pre-MPF to MPF conversion. At the end of blocking culture, oocytes were cultured with basic culture medium and were examined for GVBD at different times of the post-blocking culture. Treatment of pig oocytes with sodium vanadate counteracted the roscovitine-induced acceleration of PBMP in a concentration-dependent manner. For example, when 250 µM of sodium vanadate was used, the post-blocking GVBD completion was postponed from 12 h to 30 h (Fig. 5), a tempo similar to that observed in oocytes cultured without blocking culture (Fig. 2). Treatment with sodium vanadate also decelerated PBMP of mouse oocytes (Fig. 6).

### Epidermal growth factor in blocking medium accelerated PBMP of both pig and mouse oocytes by promoting the pre-MPF to MPF conversion

**Figure 7:**
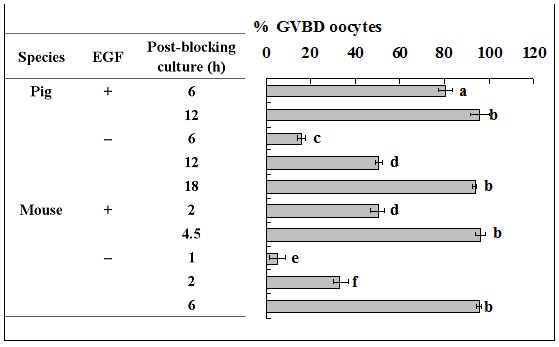
Nuclear progression of pig and mouse oocytes following blocking culture with or without EGF in the blocking medium. Whereas pig oocytes were blocked for 24–5 times with each replicate containing 15–20 oocytes, whilst for mouse oocytes, each treatment was repeated 3 times with each replicate containing some 20 oocytes. a-f: Values without a common letter in their bars differ (P<0.05).

**Figure 8:**
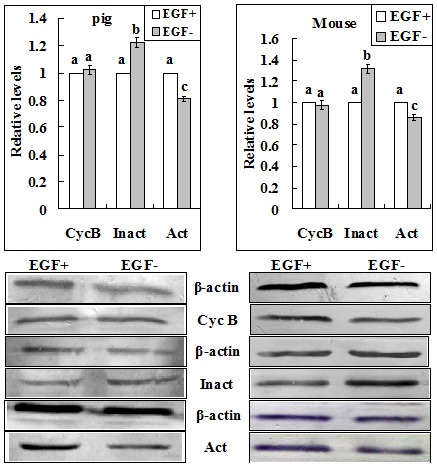
Western blot analysis for levels of Cyclin B (Cyc B), inactive (Inact) and active (Act) p-CDC2A in pig and mouse oocytes following GVBD blocking for 24 and 12 h, respectively, with (+) or without (–) EGF. a–c: Values without a common letter above their bars differ (P<0.05).

The aim of this experiment was to study the role of EGF receptor activation during blocking culture in regulating PBMP. Because the blocking medium used contained EGF, some of the pig and mouse oocytes were blocked with EGF removed in this experiment. Results showed that whereas GVBD was completed at 12 h and 4.5 h of post-blocking culture respectively in pig and mouse oocytes that had been blocked with EGF supplementation, GVBD completion was postponed to 18 h and 6 h of post-blocking culture respectively after pig and mouse oocytes were blocked without EGF (Fig. 7). Western analysis showed that EGF in blocking medium had no effect on the level of cyclin B, but its withdrawal decreased the level of active CDC2A while increasing that of inactive CDC2A (Fig. 8). This suggested that EGF in the blocking medium accelerated PBMP by promoting the pre-MPF to MPF conversion in both pig and mouse oocytes.

## Discussion

In the present study, a significant species difference in the carrying-over effect of GVBD blocking with roscovitine on PBMP was observed between mouse and pig oocytes. To study the mechanisms for this species difference, we observed the dynamics of cyclin B, the pre-MPF to MPF conversion and the activity of the EGF signaling cascade during the blocking culture. Results showed that whereas the level of cyclin B decreased significantly up to 12 h of the blocking culture in mouse oocytes, cyclin B accumulated continuously in pig oocytes during the whole period of blocking culture. Because no species difference was observed in either the pre-MPF to MPF conversion or the EGF signaling activity, it was concluded that the difference in the carrying-over effect between pig and mouse oocytes was caused mainly by the species difference in cyclin B dynamics during blocking culture with roscovitine.

Previous studies also indicated that levels of cyclin B mRNA were up regulated and its polyadenylation was increased in porcine oocytes in the presence of roscovitine [23]. Furthermore, protein synthesis and mRNA storage were observed in cattle oocytes maintained under meiotic block by roscovitine inhibition of MPF activity [22]. Then, how was the cyclin B dynamics in mouse oocytes affected by roscovitine? It could have been caused by a higher sensitivity of mouse oocytes to roscovitine compared to oocytes from other species. However, in mouse oocytes with GVBD inhibited with eCG, Han et al. [12] also observed no cyclin B synthesis, but cyclin B degradation. This suggested that in addition to roscovitine, other drugs that maintain meiotic arrest would affect cyclin B synthesis of mouse oocytes as well. Thus, why mouse oocytes are so sensitive to GVBD-inhibiting drugs will be an interesting topic for future research.

The present results demonstrated an active conversion of pre-MPF to MPF during the blocking culture with roscovitine. Thus, whereas inactive CDC2A decreased, the active CDC2A increased continuously up to 48 h of blocking culture of pig oocytes. A similar negative correlation between active and inactive CDC2A was also observed in mouse oocytes from 12 h to 24 h of the blocking culture. Treatment with sodium vanadate, a well-known inhibitor blocking the pre-MPF to MPF conversion [24], decelerated PBMP significantly in both mouse and pig oocytes. Furthermore, EGF in the blocking medium was found to accelerate PBMP in both species by promoting the pre-MPF to MPF conversion. A conversion of inactive pre-MPF to active MPF has been reported in aging porcine oocytes [24]. It is controlled by a complex cascade of phosphorylation and de-phosphorylation events. For instance, the phosphorylation of CDC2A on T14 and Y15 by the Myt1 and Wee1 kinases produces pre-MPF [15], and for full activation, CDC2A needs to be phosphorylated on T161 by CAK and dephosphorylated on T14 and Y15 by cdc25 phosphatase [16]. Roscovitine specifically interferes with MPF activity by blocking the ATP binding pocket of CDC2A [25] where T14 and Y15 residues are localized [26]. Therefore, roscovitine may prevent pre-MPF formation by affecting the phosphorylation of CDC2A on T14 and Y15, but it will not affect the pre-MPF to MPF conversion because it does not interfere with dephosphorylation of CDC2A on T14 and Y15. In fact, more inactive CDC2A was found in immature prepubertal goat oocytes than in roscovitine-treated and IVM oocytes, and the MPF activity was higher in oocytes exposed to roscovitine for 24 h than oocytes at collection time [27].

The present results indicated that active CDC2A accumulated continuously during blocking culture of pig oocytes. In addition, a dramatic decrease in inactive CDC2A along with the same extent of increase in active CDC2A were observed in pig oocytes at 48 h of blocking culture. Although this may suggest a burst of pre-MPF to MPF conversion when oocytes began GVBD (because some 60% of the oocytes underwent GVBD by this time), it may also suggest the possibility for a direct phosphorylation of CDC2A on T161 to activate MPF because an increase in cyclin B was also observed by this time. Although it has been reported that CAK exhibits a stronger affinity for cyclin-associated CDC2s than for monomeric CDC2s [28], a “monomeric” CAK activity has been detected in human cells [29]. If such an enzyme were expressed, monomeric CDC2A would be phosphorylated on T161 in the cytoplasm prior to its association with newly synthesized cyclin B and prior to its inactivation by the membrane-associated Myt1 kinase. The activation of free CDC2A already phosphorylated on T161 dependent on cyclin B and could contribute to the generation of the MPF kinase activity threshold required to initiate MPF amplification [30]. Cyclin B could induce the phosphorylation of CDC2A on T161, stabilize the low level phosphorylation of CDC2A and drive the equilibrium to the phosphorylated form [31]. In marine nemertean worm egg, active MPF, which has phosphorylated T161 and non-phosphorylated Y15 on CDC2A, was at low levels in immature eggs and at high levels in mature eggs, and inactive MPF (high p-Y15, low p-T161) was high in immature eggs and low in mature eggs [32]. T161 phosphorylation of CDC2A was very low until 12 h and increased at 18 h. This increase of T161 phosphorylation corresponded well with the start of GVBD. Furthermore, CDK7 over expression accelerated meiotic resumption of porcine oocytes by acceleration of MPF activation through T161 phosphorylation of CDC2A [33]. However, although the above literature suggested that the phosphorylation of T161 in CDC2A was not affected by the presence of roscovitine, the possibility could not be excluded that roscovitine did not totally block CDC2A phosphorylation while keeping the active MPF under the threshold level for GVBD through unknown mechanisms.

This study showed that EGF in the blocking medium accelerated PBMP of both pig and mouse oocytes. Our western analysis indicated that EGF accelerated PBMP not by increasing cyclin B but by promoting the pre-MPF to MPF conversion. Although several studies observed that EGF accelerated oocyte meiotic progression in the process of normal IVM [34]–[36], studies are few on its effect on PBMP following GVBD blocking. Roscovitine was able to inhibit MPF activities at a lesser extent when bovine [22] and horse [37] oocytes were cultured in the presence of EGF. Tsuji et al. [38] demonstrated that NPPC/NPR2 signaling was essential for oocyte meiotic arrest and the activated EGFR signaling reduced NPPC inhibition of meiotic resumption by the down regulation of NPPC mRNA, which subsequently depleted oocyte cGMP provided from cumulus cells, thereby allowing oocyte meiosis to resume through lowered cAMP levels. Wang et al. [39] reported that EGF receptor signaling induced meiotic resumption by elevating calcium concentrations of cumulus cells to decrease NPR2 guanylyl cyclase activity and cGMP levels. We thus propose that during the blocking culture with roscovitine, the addition of EGF may promote the pre-MPF to MPF conversion of oocytes by reducing the PKA activity through decreasing cAMP levels.

In conclusion, we have studied the species difference and the signaling events leading to the carrying-over effect of GVBD blocking on PBMP. Results indicated that (a) a significant species difference in PBMP was observed between mouse and pig oocytes following GVBD blocking with roscovitine; (b) the EGF signaling cascade was active during GVBD blocking with roscovitine, which accelerated PBMP by promoting the pre-MPF to MPF conversion; (c) no species difference was observed in either the pre-MPF to MPF conversion or the EGF signaling activity during the blocking culture. It was thus concluded that the significant difference in PBMP observed between mouse and pig oocytes was caused mainly by the species difference in cyclin B dynamics during the blocking culture. Data obtained on the molecular events during GVBD blocking are important not only for efforts to improve the quality of IVM oocytes by blocking GVBD and allowing more time for ooplasmic maturation but also for procedures aimed at regulating gene expression in maturing oocytes prior to GVBD.

## Materials and Methods

### Ethics Statement

Mouse care and use were conducted exactly in accordance with the guidelines and approved by the Animal Research Committee of the Shandong Agricultural University, P. R. China (Permit number: 20010510). According to the guidelines of the committee, the animal handling staff (including each post-doc, doctoral or masters student) must be trained before using animals. Mice must be housed in a temperature-controlled room with proper darkness-light cycles, fed with a regular diet, and maintained under the care of the Experimental Animal Center, Shandong Agricultural University College of Animal Science and Vet Medicine. In the present study, mice were sacrificed by cervical dislocation. The only procedure performed on the dead animals was the collection of oocytes from the ovaries.

All chemicals and reagents were purchased from Sigma Chemical Company (St. Louis, MO, USA) unless otherwise specified.

### Animals and oocyte recovery

Mice of the Kunming breed were kept in a room with 14/10-hour light-dark cycles, with the dark cycle starting at 8 P.M. Female mice, 8 to 10 weeks after birth, were killed 48 h after injection with equine chorionic gonadotropin (eCG, 10 IU/mouse) and the large follicles on the ovary were punctured in M2 medium to release cumulus-oocyte- complexes (COCs). Only COCs with more than three layers of unexpanded cumulus cells and containing oocytes of >75 µm in diameter and with a homogenous cytoplasm were selected for use.

Porcine ovaries were collected at the Feicheng slaughterhouse of Yinbao Food Corporation Ltd. (Tai-an city, China) and delivered to the laboratory within 3 h after slaughtering in a thermos bottle with sterile saline maintained at 37°C. The COCs were recovered by aspirating the healthy follicles of 3–6 mm in diameter using a syringe. Oocytes recovered were washed four times in Dulbecco’s phosphate-buffered saline (D-PBS) and only COCs with uniform ooplasm and compact cumulus were chosen for further treatment.

### In vitro culture of oocytes

#### Culture media and preparation

The basic medium was TCM-199 (Gibco, Grand Island, New York, USA) supplemented with 0.91 mM sodium pyruvate, 1.0 g/l PVA, 3.05 mM D-glucose, 75 µg/ml penicillin G, 50 µg/ml streptomycin, 0.05 IU/ml FSH and 10 ng/ml EGF. The blocking medium used for GVBD inhibition was made of the basic medium supplemented with 100- and 25-µM roscovitine for mouse and pig oocytes, respectively. To study the effect of EGF, EGF (10 ng/ml) was removed from the blocking medium. Depending on experimental design, 5 µg/ml cycloheximide (CHX) or different concentrations of sodium vanadate were added to the blocking medium. To make stock solutions, CHX (1 mg/ml), sodium vanadate (100 mM), and EGF (100 µg/ml) were dissolved in water, while roscovitine (20 mM) was dissolved in dimethyl sulfoxide (DMSO). All of the stock solutions were stored frozen in aliquots at −20°C and diluted to the desired concentrations before use.

#### Blocking culture

Both mouse and pig COCs were cultured in drops of 100-µl blocking medium, covered with mineral oil, under 5% CO_2_ in humidified air. Whereas mouse oocytes were blocked at 37°C with each drop containing 20–25 COCs, pig oocytes were cultured at 38.5°C with each drop containing 15–20 COCs.

#### Post-blocking culture

After blocking culture, oocytes were washed three times in M2 (mouse oocytes) or D-PBS (pig oocytes) medium and cultured in the basic medium for different times to examine the meiotic progression.

### Observation for meiotic progression

At the end of post-blocking culture, COCs were freed of cumulus cells mechanically by pipetting with a thin pipette. Whereas the mouse cumulus-denuded oocytes were examined under a phase contrast microscope for the status of GV, the pig cumulus-denuded oocytes were examined under a fluorescence microscope following staining with 10-µg/ml Hoechst 33342.

### Western blot analysis

One hundred and fifty cumulus-free oocytes were placed in a 1.5 ml microfuge tube containing 20-µl sample buffer (20 mM Hepes, 100 mM KCl, 5 MgCl_2_, 2 mM DTT, 0.3 mM PMSF, 3 µg/ml leupetin, PH 7.5) and frozen at –80°C until use. NaF (10 mM) was added to the sample buffer when p-CDC2A was assayed. For protein extraction, 5 µl of 5× SDS-PAGE loading buffer was added to each tube and the tubes were heated at 100°C for 5 min. The total proteins were separated on a 12% polyacrylamide gel by SDS-PAGE and transferred electrophoretically onto PVDF membranes. The membranes were washed twice in TBST (150-mM NaCl, 2-mM KCl, 25-mM Tris, 0.05% Tween-20, pH 7.4) and blocked with TBST containing 3% BSA at room temperature for 30 min. The membranes were then incubated at 4°C overnight with primary antibodies at a dilution of 1∶1000 in 3% BSA -TBST. After being washed three times in TBST (5 min each), the membranes were incubated for 1 h at 37°C with second antibodies diluted to 1∶1000 in 3% BSA -TBST. After three washings in TBST, the membranes were detected by a BCIP/NBT alkaline phosphatase color development kit (Beyotime Institute of Biotechnology, China). The relative quantities of proteins were determined with an Image-Pro Plus software by analyzing the sum density of each protein band image. The quantity values of freshly collected GV oocytes were arbitrarily set as 100% and the other values were expressed relative to this activity. β-actin was assayed for internal controls.

The primary antibodies used included mouse anti-cyclin B (Santa Cruz, sc-245), rabbit anti-phospho-Cdc2 (Thr14/Tyr15) (Santa Cruz, sc-12340-R), rabbit anti-phospho-Cdc2 (Thr161) (Santa Cruz, sc-12341), rabbit anti-Cdc2 (Santa Cruz, sc-53), and mouse anti-β-actin (CWBIO, cw0096). The secondary antibodies included horse anti-mouse IgG AP conjugated (ZSGB-Biotechnology, ZB-2310) and goat anti-rabbit IgG AP conjugated (CWBIO, cw0111).

### Data analysis

There were at least three replicates for each treatment. Percentage data were arc-sine transformed and analyzed with ANOVA; a Duncan multiple comparison test was used to locate differences. The soft ware used was SPSS (Statistics Package for Social Science). Data were expressed as mean ± SE and P<0.05 was considered significant.
